# Antibacterial and Antifungal Activity of *Holothuria leucospilota* Isolated From Persian Gulf and Oman Sea

**DOI:** 10.5812/jjm.8708

**Published:** 2014-01-01

**Authors:** Neda Adibpour, Farhad Nasr, Fatemeh Nematpour, Arash Shakouri, Abdolghani Ameri

**Affiliations:** 1 Marine Pharmaceutical Sciences Research Center, School of Pharmacy, Ahvaz Jundishapur University of Medical Sciences, Ahvaz, IR Iran; 2 Department of Medicinal Chemistry, School of Pharmacy, Ahvaz Jundishapur University of Medical Sciences, Ahvaz, IR Iran; 3 School of Marine Sciences, Chabahar Maritime University, Chahbahar, IR Iran; 4 Department of Food and Drug Control, School of Pharmacy, Ahvaz Jundishapur University of Medical Sciences, Ahvaz, IR Iran

**Keywords:** *Holothuria leucospilota*, Anti-Infective Agents, Persian Gulf

## Abstract

**Background:**

Emergence of antimicrobial resistance toward a number of conventional antibiotics has triggered the search for antimicrobial agents from a variety of sources including the marine environment.

**Objectives:**

The aim of this study was to evaluate the antimicrobial potential of *Holothuria leucospilota* from Qeshm and Kharg Islands against some selected bacteria and fungi.

**Materials and Methods:**

In this investigation, sea cucumbers from two coastal cities of Persian Gulf were collected in March and May 2011 and identified by the scale method according to the food and agriculture organization of the United Nations. Antibacterial activity of hydroalcoholic extracts of the body wall, cuvierian organs and coelomic fluid, methanol, chloroform, and n-hexane extracts of the body wall were evaluated by the spot test. In addition, their antifungal activity was assessed by the broth dilution method.

**Results:**

The displayed effect was microbiostatic at concentrations of 1000 and 2000 µg/mL rather than microbicidal. The highest activity of hydroalcoholic extracts was exhibited by body wall, cuvierian organs and coelomic fluid against *Escherichia coli*, *Salmonella typhi*, *Staphylococcus aureus* and *Pseudomonas aeruginosa;* *Aspergillus niger*, *A. fumigatus*, *A. flavus* and *A. brasilensis*. However, none of the methanol, chloroform and n-haxane extracts showed appreciable effects against *Shigella dysenteriae*, *Proteus vulgaris*, *Bacillus cereus*, *S. epidermidis* and *Candida albicans*. Moreover, cuvierian organs did not possess any antifungal potential.

**Conclusions:**

Our data indicated that water-methanol extracts from the body wall of *H. leucospilota* possess antibacterial and antifungal activity. However, additional and in-depth studies are required to isolate and identify the active component(s).

## 1. Background

Emergence of antimicrobial resistance toward a number of conventional antibiotics has stimulated the search for antimicrobial agents from a variety of sources including the marine environments. Sea cucumbers are echinoderms from the class *Holothuroidea*. In Vietnamese traditional medicine, sea cucumbers had been used as tonics and delicacies (1). In Malaysia, different species of sea cucumbers are used to relieve pain and skin irritations and treat eczema and arthritis (2). Antimicrobial activities of several species of echinoderms from the Gulfs of California, Mexico and Caribbean Islands and the Coast of Norway have been reported (3-5).

Various antimicrobial components including steroidal glycosides (6), polyhydroxylated sterols (7), naphthoquinone pigments (8), lysozymes (9, 10), complement-like substances (11) and antimicrobial peptides (12) have been isolated from the sea cucumbers. Additionally, several holostane-type triterpene glycosides (from *Holothuria fuscocinerea*) (13) and three new cytotoxic triterpene glycosides (from *Mensamaria intercedens* Lampert) displaying broad range of antibacterial, antifungal and cytotoxic activity (14), have been isolated. 

Although the focus of study on marine organisms such as echinoderms and holothuroids is increasing, information regarding exploitation and fishing techniques in Iran is scanty and recent. However, due to the expansive coastal area of Iran, most coastal cities have some species of Holothurians (15). Since sea cucumbers are not popular in Iran, out of 1400 globally-recorded (16) Holothurian species, so far only 20 have been recorded in Iran (17). The most harvested sea cucumber in Iran is the sandfish, *H*.* scabra* or Khiar Daryaei (as it is called in the local language), harvest of which began in 2004 at Qeshm Island (18).

## 2. Objectives

Since the published data about the antimicrobial activity of sea cucumbers in Persian Gulf and Oman Sea is very scarce, the aim of this study was to evaluate the antimicrobial potential of the sea cucumber, *H*.* leucospilata*, collected from Qeshm and Kharg islands in Persian Gulf and Oman Sea.

## 3. Materials and Methods

### 3.1. Collection of Samples

Sea cucumbers were harvested freshly from fixed sites of two coastal cities of Kharg and Qeshm in the Persian Gulf and also from the rocky beach of Tis village in Oman Sea during March and May of 2011. Samples were rinsed with distilled water to remove debris and foreign particles. All samples were identified according to the food and agriculture organization of the United Nations (19). Following identification, all samples were maintained at -20°C until the usage time.

### 3.2. Processing of Samples

Initially, body wall, cuvierian organs, and coelomic fluid were separated. Then, each part was cut into several pieces and dried at room temperature in the dark, then milled to a fine powder.

### 3.3. Extraction of Samples

Crude methanol extracts of *H. leucospilota* were prepared by maceration of different body parts in appropriate amounts of methanol-water (50:50), mixing, and maintenance for 16 hours. Then, the mixture was filtered and the process was repeated for the second time. Finally, the two portions were pooled together. The filtrate was concentrated to dryness by rotary evaporation. The obtained powder was subjected to extraction with n-hexane, chloroform and ethyl acetate and then all the extracts were dried by flash evaporation.

### 3.4. Test Microorganisms

Test bacteria used in the study included standard strains of *Shigella dysenteriae* (ATCC 13313), *Proteus vulgaris* (ATCC 29905), *Escherichia coli* (ATCC 8739), *Salmonella typhimurium* (ATCC 19430), *Pseudomonas aeruginosa* (ATCC 9027), *Staphylococcus aureus* (ATCC 29737), *S*.* epidermidis* (ATCC 12229), and *Bacillus ceureus* (ATCC 11778). Fungi included clinical isolates of *Aspergillus flavus*, *A*.* fumigatus*, *A*.* niger*, *A*.* nidulans*, *A*.* restatus* and standard strains of *A*.* brasiliensis *(ATCC 9029) and *Candida albicans *(ATCC 10231). All standard ATCC microorganisms were procured from the institute for collection and maintenance of pathogenic and industrial bacteria and fungi of Iranian research organization for science and technology (IROST), Tehran, Iran.

### 3.5. Antibacterial Assay

Various extracts of different body parts were assayed against the test bacteria using the spot test (20). Initially, various concentrations (2000 - 40000 µg/mL) of different dried extracts were prepared in sterile distilled water, then 1 mL of each extract was transferred to a sterile petri plate, 19 mL of molten Meuller-Hinton agar medium was added, the contents of plates were gently rotated and allowed to solidify to give the concentration range of 100 - 2000 µg/mL. Microbial suspensions for each test microorganism were prepared and adjusted according to 0.5 McFarland standard. Ten microliters of each tested bacteria was deposited on the surface of each plate. Bacteria were incubated at 37°C for 24 hours. At the end of the incubation period, plates were examined for growth and minimal inhibitory concentration of each extract was determined. All assays were repeated two times.

### 3.6. Antifungal Assay

Before starting the antifungal assay, each test strain was cultured on Sabouraud's dextrose agar and incubated for 1 - 2 days (*C. albicans*) or 2 - 3 days (*Aspergillus* spp.) at 37°C. For *Aspergillus* spp, suspensions of each strain in 3 mL of sterile distilled water containing 500 ppm Tween 80 (Merck, Germany) were prepared by vortex mixing. After filtration through a sterile celite to remove the hyphal fragments and residual agar, the starting inocula were adjusted to 10^4^ CFU/mL by sterile distilled water using spectrophotometry at 530 nm. Preparation of *C. albicans* was done in the same manner without using Tween 80 (21, 22).

Stock solutions of the extracts were prepared in sterile water. The resulting solutions were progressively double diluted with the test medium (RPMI 1640 (Sigma, USA) supplemented with L-glutamin without sodium bicarbonate and buffered to pH 7 with 0.165 M (35.54 g/L) morpholinesulfonic acid (MOPS)) to give the final concentration range of 100 - 2000 µg/mL. Blanks were prepared in the test medium using the same quantities of water, but without the test extracts. One mililiter of each strain was mixed with 1 mL of media-containing test extracts or in 5-mL culture tubes and tubes were incubated at 37°C for 48 hours. The MIC values were determined as the lowest concentration of the test extract with no visible growth.

## 4. Results

Hydroalcoholic extract (50%) of *H. leucospilota *exhibited antibacterial and antifungal activity against some of the test microorganisms (Table 1). Results with antibacterial effects showed microbiostatic effects rather than microcidal. The highest activity was exhibited by the body wall, Cuvierian organs, and coelomic fluid against *E. coli*, *S. typhi*, *S. aureus *and *P. aeruginosa*. coelomic fluid also inhibited the growth of these bacteria, but at a slower rate. Hydoalcoholic extract of the three examined body tissues did not inhibit the growth of *S. dysenteriae*, *P. vulgaris*, *B. cereus *and *S. epidermidis*. 

Table 2 depicts the results of all extracts examined for antifungal activity. As presented in this Table, cuvierian extracts did not inhibit the fungal growth even at a higher concentration (2000 µg/mL). However, the body wall and coelomic fluid inhibited the growth of all fungi at both concentrations; nonetheless, body wall extract at 1000 µg/mL did not inhibit the growth of *A. niger*. 

**Table 1.** Antimicrobial Activity of Various Extracts of *H *. *leucospilata *Against Different Species of Bacteria

Test Organism	Extracts, µg/mL					
	Body Wall		Cuvierian Organs		Coelomic Fluid	
	2000	1000	2000	1000	2000	1000
***B. cereus***	- ^a^	-	-	-	-	-
***S.epidermidis***	-	-	-	-	-	-
***S. aureus***	+ ^b^	+	+	+	+	+
***S. dysenteriae***	-	-	-	-	-	-
***S. typhi***	+	+	+	+	+	+
***P. proteus***	-	-	-	-	-	-
***E.coli***	+	+	+	+	+	+
***P.aeruginosa***	+	+	+	+	+	+

^a ^-: no activity.

^b ^+: antimicrobial activity.

**Table 2.** Antimicrobial Activity of Various Extracts of *H *. *leucospilata *Against Different Species of Fungi

Test Organism	Extracts, µg/mL					
	Body Wall		Cuvierian Organs		Coelomic Fluid	
	2000	1000	2000	1000	2000	1000
***C. albicans***	- ^a^	-	-	-	-	-
***A. niger***	+ ^b^	+	-	-	+	-
***A. fumigatus***	+	+	-	-	+	+
***A. flavus***	+	+	-	-	+	+
***A. brasilensis***	+	-	-	-	+	+

^a ^-: no activity.

^b ^+: antimicrobial activity.

No significant difference of antibacterial and antifungal activity was observed between the *H. leucospilota* collected from Persian Gulf and Oman Sea.

## 5. Discussion

Hydroalcoholic (50%), n-hexane, chloroform, and methanol extracts from different tissues and organs of the sea cucumber, *H. leucospilota,* were screened for antimicrobial activities against an array of Gram positive and Gram negative bacteria as well as fungi and molds. From all extracts prepared with different solvents, only the hydroalcoholic extracts of the body wall, Cuvierian organs, and coelomic fluid exhibited antibacterial and antifungal activities in vitro. Previous studies reported antimicrobial activities from various species of echinoderms (2, 4, 6). In most of the studied species, the whole bodies or body walls were tested. Other studies reported antimicrobial activities of egg extracts of *Paracentrotus lividus* (23) and *Marthasterias glacialis* (10). In the latter study, the active compound was reported to be a lysozyme.

Our study indicates that the component(s) responsible for antimicrobial activity appear(s) to be concentrated mainly in the body wall and coelomic fluid; but little or no activity was observed in the cuvierian organs since there was moderate activity toward some species of bacteria and none against any of the tested fungi and molds. The methanol-water extracts of tissues/organs exhibited antimicrobial activities against some selected species of Gram positive and Gram negative bacteria as well as fungi, suggesting that multiple factors are responsible for these activities. Thus, additional in-depth chemical analyses are required for isolation and purification of the active compound(s) as well as identification of their chemical nature and evaluation of their potential strength for novel drugs.

In conclusion, the body wall and coelomic fluid of *Holothuria leucospilota* collected from Persian Gulf and Oman Sea showed weak antibacterial and antifungal effects against few species of human pathogenic bacteria and fungi undertaken in this study.
